# Hepatitis C Virus Exploits Death Receptor 6-mediated Signaling Pathway to Facilitate Viral Propagation

**DOI:** 10.1038/s41598-017-06740-9

**Published:** 2017-07-25

**Authors:** Trang T. D. Luong, Giao V. Q. Tran, Dong-Jo Shin, Yun-Sook Lim, Soon B. Hwang

**Affiliations:** 1 National Research Laboratory of Hepatitis C Virus and Ilsong Institute of Life Science, Hallym University, Anyang, South Korea; 2 Present Address: Institut Pasteur Korea, Bundang-gu, Seongnam Republic of Korea

## Abstract

The life cycle of hepatitis C virus (HCV) is highly dependent on host proteins for virus propagation. By transcriptome sequencing analysis, we identified host genes that were highly differentially expressed in HCV-infected cells. Of these candidates, we selected Death receptor 6 (DR6) for further characterization. DR6 is an orphan member of the tumor necrosis factor receptor superfamily. In the present study, we demonstrated that both mRNA and protein levels of DR6 were increased in the context of HCV replication. We further showed that promoter activity of DR6 was increased by HCV infection. By employing promoter-linked reporter assay, we showed that HCV upregulated DR6 via ROS-mediated NF-κB pathway. Both mRNA and protein levels of DR6 were increased by NS4B or NS5A. However, NS5A but not NS4B specifically interacted with DR6. We showed that HCV modulated JNK, p38 MAPK, STAT3, and Akt signaling pathways in a DR6-dependent manner. Interestingly, Akt signaling cascade was regulated by protein interplay between DR6 and NS5A. Silencing of DR6 expression resulted in decrease of infectious HCV production without affecting viral entry, replication, and translation. Together, these data indicate that HCV modulates DR6 signaling pathway for viral propagation and may contribute to HCV-mediated pathogenesis.

## Introduction

Hepatitis C virus (HCV) modulates host cellular signaling pathways and immune responses to maintain persistent infection^1, 2^. The prominent feature of HCV infection is the development of chronicity in HCV-infected patients. Consequently, HCV infection often causes extensive fibrosis, cirrhosis, and hepatocellular carcinoma (HCC)^3^. HCV, a member of the *Flaviviridae* family, is a positive-sense, single-stranded RNA virus. The 9.6-kb HCV genome encodes a long precursor polyprotein, which is sequentially processed into 3 structural proteins (Core, E1, and E2) and 7 nonstructural proteins (p7 and NS2 to NS5B)^4^. Approximately 3% of the world’s population is infected with HCV. However, there is no prophylactic vaccine for HCV yet. Recent development of direct-acting antiviral (DAAs) is highly successful in the treatment of certain genotypes of HCV. However, there are still many issues, including high cost of drugs, genotypic differences in cure rates, and occurrence of drug resistant-associated variants. Since HCV life cycle is highly dependent on host factors, identification of host factors involved in HCV propagation could be an alternative way to develop host-targeting antivirals with a high genetic barrier to resistance.

Death receptor (DR6, also known as TNFRSF21) is a tumor necrosis factor related death receptor family which contains a death domain^5^. DR6 is encoded by *TNFRSF21* gene. It consists of 655 amino acid residues and its predicted size is 72-kDa protein. DR6 protein has three major domains: an N-terminal extracellular cysteine-rich domain, a transmembrane domain, and a C-terminal cytoplasmic death-domain^6^. N-terminal domain of DR6 is responsible for posttranslational modification^7^ and C-terminal domain of DR6 regulates apoptotic signaling^6^. Human DR6 contains six potential N-linked glycosylation sites and multiple putative O-linked oligosaccharide chains. Glycosylation pattern of DR6 varies on cell types and confers both structural and functional properties^7, 8^. DR6 has been involved in various cellular events, including apoptosis^6, 8^ and tumor growth^9^. It has been also reported that overexpression of DR6 is involved in JNK^10^, p38 MAPK, and STAT3 signaling pathways^9^. As a member of the superfamily of TNF receptor, DR6 is upregulated by TNF-α via NF-κB activation in prostate tumor cell lines^10^. Similarly, activation of NF-κB and NF-AT signaling pathways transiently enhance DR6 expression in both activated human CD4^+^ and CD8^+^ T cells^11^. However, functional involvement of DR6 in viral infection has not been demonstrated yet.

Using RNA-Seq analysis, we recently identified 30 host genes which were upregulated in HCV-infected cells^12^. In the present study, we demonstrated that HCV infection upregulated DR6 expression via ROS-mediated NF-κB pathway. We also showed that HCV modulated JNK, p38 MAPK, STAT3, and Akt signaling pathways in a DR6-dependent manner. Of note, NS5A specifically interacted with DR6 and regulated Akt signaling cascade. Moreover, DR6 was required for the production of infectious HCV. Collectively, these data suggest that DR6 is not only required for HCV propagation but also involved in HCV-associated pathogenesis.

## Results

### HCV upregulates DR6 expression

**Figure 1:**
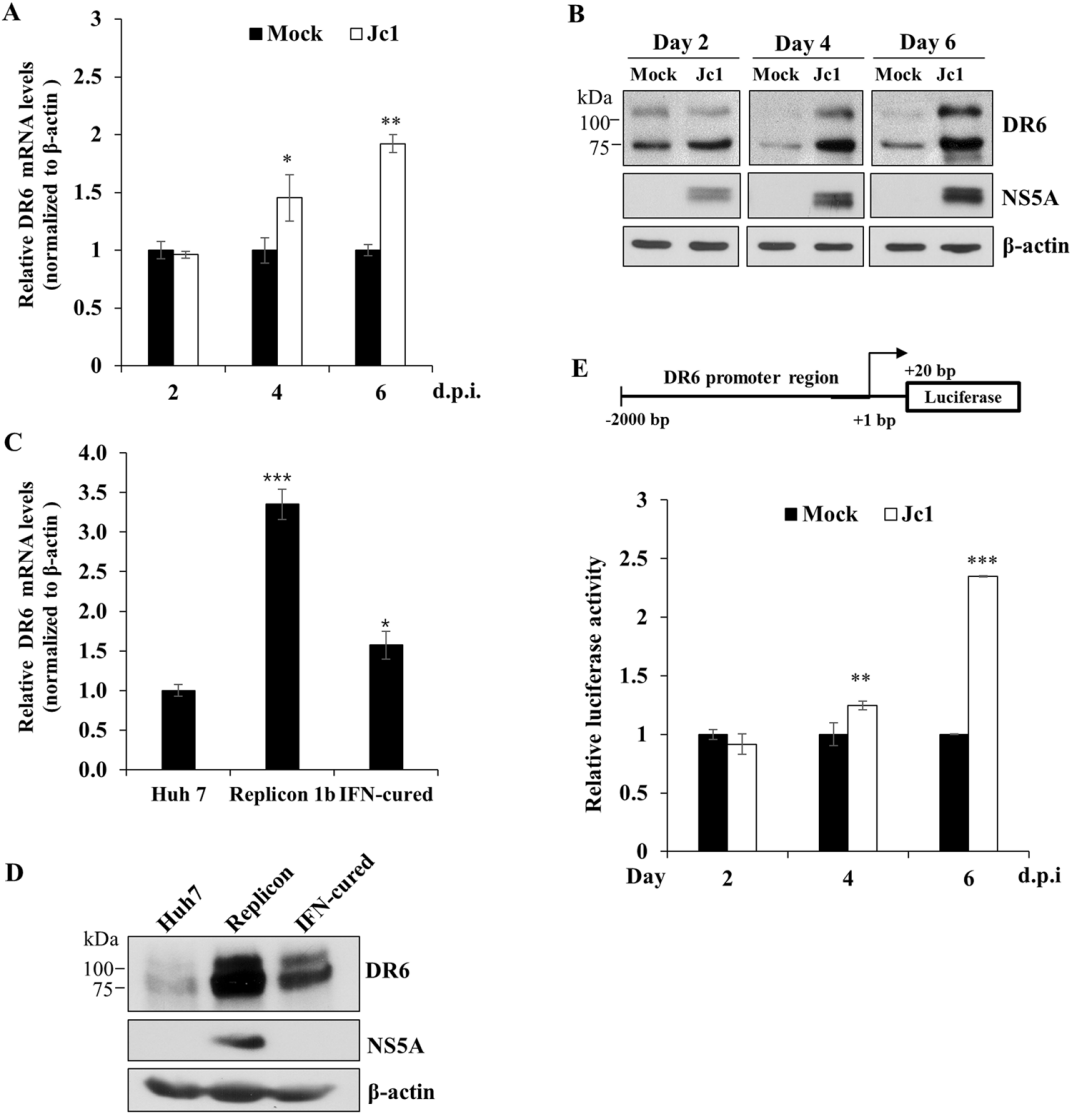
HCV upregulates DR6 expression levels. (**A**) Huh7.5 cells were either mock-infected or infected with HCV Jc1 for 4  h and then mRNA levels of DR6 were analyzed by qRT-PCR at the indicated time points. d.p.i., days postinfection. (**B**) Huh7.5 cells were either mock-infected or infected with HCV Jc1 for 4 h. Total cell lysates were harvested at the indicated time points and were immunoblotted with the indicated antibodies. (**C**) mRNA levels of DR6 in Huh7 cells, IFN cured cells, and replicon cells derived from HCV genotype 1b were determined by qRT-PCR. (**D**) Protein levels of DR6 in Huh7 cells, IFN cured cells, and replicon cells derived from genotype 1b were immunoblotted with the indicated antibodies. (**E**) (Upper panel) Schematic illustration of DR6-luc promoter reporter construct. (Lower panel) Huh7.5 cells were either mock-infected or infected with Jc1 for 4 h and then further transfected with DR6-luc promoter reporter plasmid. Cells were harvested and luciferase activities were determined at the indicated time points. Data from two independent experiments were quantified. The asterisks indicate significant differences (*P < 0.05; **P < 0.01; ***P < 0.001) from the value for the control.

We previously performed next generation sequencing (RNA-Seq) and identified 30 genes that were highly differentially expressed in HCVcc-infected cells^12^. In the present study, we selected DR6, a member of the TNF receptor family, for further characterization. We demonstrated that mRNA level of DR6 was significantly increased in HCV-infected cells compared with mock-infected cells (Fig. 1A). Consistently, protein expression level of DR6 was gradually increased up to day 6 postinfection (Fig. 1B). Protein expression patterns of DR6 vary among cell lines^8^. Our data showed that two forms of DR6 protein, ~75 kDa and ~110 kDa, were expressed in Huh7 and Huh7.5 cells (Supplementary Fig. S1), which is consistent with the previous report^7^. We also demonstrated that both mRNA (Fig. 1C) and protein (Fig. 1D) levels of DR6 were prominently augmented in HCV subgenomic replicon cells compared with those in parental Huh7 and IFN-cured cells. We also observed that mRNA level of DR6 was significantly increased in primary human hepatocytes (Supplementary Fig. S2). To further investigate whether HCV upregulated the transcriptional level of DR6, Huh7 cells were transfected with a luciferase reporter construct consisting of nt −2000 to +20 of the DR6 promoter (Fig. 1E, upper panel) and then cells were either mock-infected or infected with Jc1. We demonstrated that promoter activities of DR6 were also gradually increased during the course of HCV infection compared to mock-infected cells (Fig. 1E, lower panel). These data suggest that HCV upregulates DR6 expression.

### HCV upregulates DR6 via ROS-mediated NF-κB pathway

**Figure 2:**
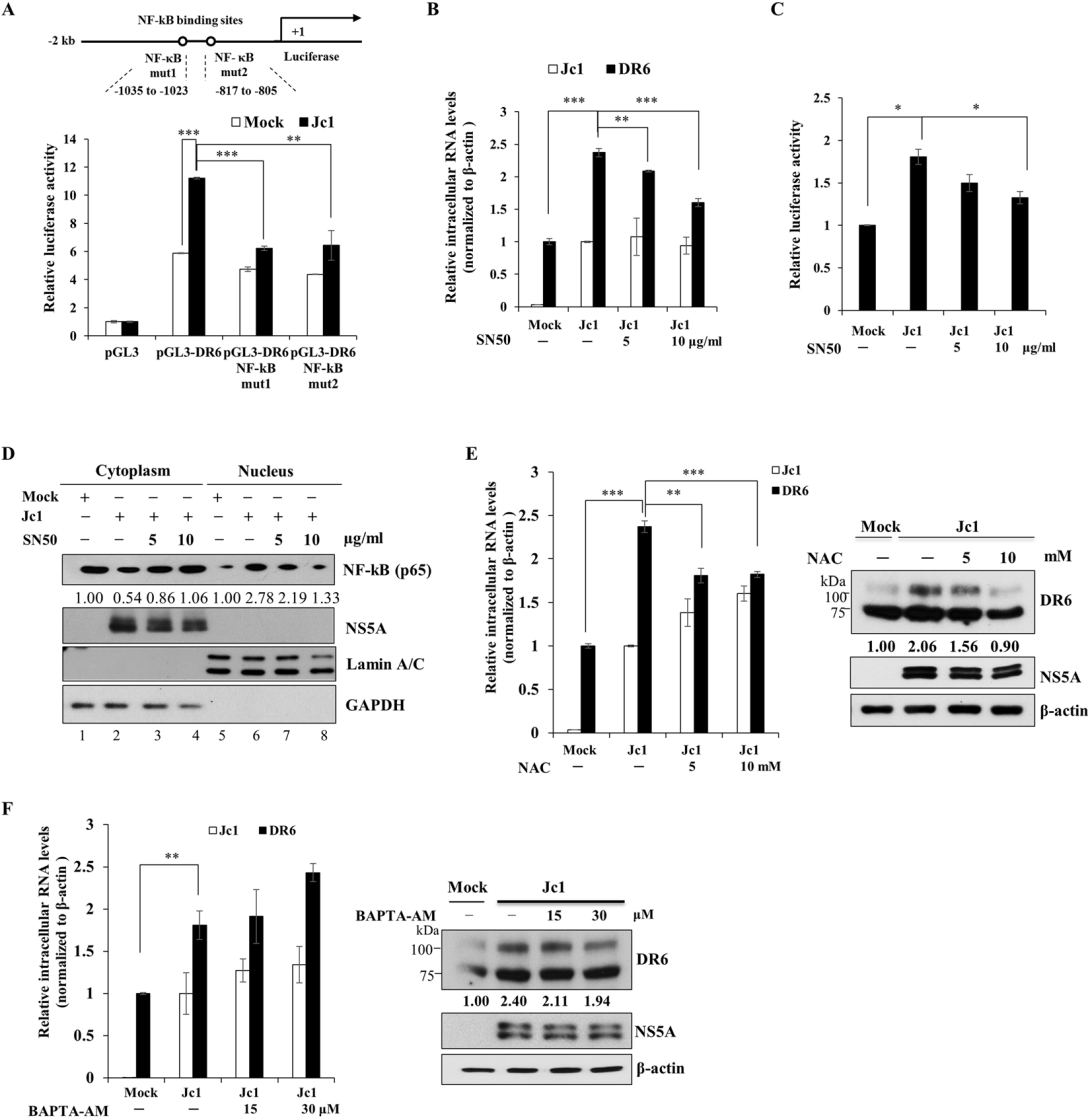
HCV promotes DR6 expression via ROS-mediated NF-κB activation. (**A**) (Upper panel) Shematic diagram of DR6-luc promoter construct with mutations in NF-κB binding sites. (Lower panel) Huh7.5 cells were either mock-infected or infected with HCV Jc1 for 4 h. At 48 h postinfection, cells were transfected with pGL3 vector, wild-type or mutants of DR6 promoter. At 48 h after transfection, cells were harvested and luciferase activities were determined. Data from two independent experiments were quantified. The asterisks indicate significant differences (*P < 0.05, **P < 0.01; ***P < 0.001) from the value for the control. (**B**) Huh7.5 cells were either mock-infected or infected with HCV Jc1 for 4 h. At 48 h postinfection, cells were either left untreated or treated with increasing amounts of SN50. At 48 h after inhibitor treatment, mRNA levels of DR6 were determined by qRT-PCR. (**C**) Huh7.5 cells were infected with Jc1 for 4 h. At 48 h postinfection, cells were either left untreated or treated with increasing amounts of SN50. At 48 h after inhibitor treatment, DR6 promoter activities were analyzed. (**D**) Huh7.5 cells were either mock-infected or infected with Jc1 for 4 h. At 48 h postinfection, cells were either left untreated or treated with increasing amounts of SN50. At 48 h after inhibitor treatment, cytosolic and nuclear fractions were subjected to immunoblot analysis to determine NF-κB level. GAPDH and Lamin A/C were used as cytoplasmic and nuclear marker, respectively. (**E**) Huh7.5 cells were either mock-infected or infected with Jc1 for 72 h. Cells were either left untreated or treated with increasing amounts of antioxidant agent NAC. At 24 h after NAC treatment, both mRNA (left panel) and protein (right panel) levels of DR6 were analyzed by qRT-PCR and immunoblot analysis, respectively. (**F**) Huh7.5 cells were either mock-infected or infected with Jc1 for 72 h. Cells were either left untreated or treated with increasing amounts of intracellular Ca^2+^ chelator, BAPTA-AM. At 24 h after BAPTA-AM treatment, both mRNA (left panel) and protein (right panel) levels of DR6 were analyzed by qRT-PCR and immunoblot analysis, respectively. Protein band intensities were analyzed by using ImageJ.

It has been previously reported that expression of DR6 is regulated by TNFα via NF-κB signaling in LnCaP and HEK293T cells^10^. We therefore investigated whether HCV-induced upregulation of DR6 was also mediated through NF-κB activation. For this purpose, we constructed two mutants bearing mutations in putative NF-κB binding sites in DR6 promoter (Fig. 2A, upper panel). Huh7.5 cells were either mock-infected or infected with Jc1 and then cells were transfected with either wild-type or mutants of DR6 promoter. We showed that HCV-induced DR6 promoter activities were abrogated in both mutants, indicating that HCV upregulated DR6 expression via NF-κB activation (Fig. 2A, lower panel). To corroborate this effect of NF-κB activation on DR6, Huh7.5 cells were treated with increasing concentrations of a NF-κB inhibitor, SN50. As expected, SN50 inhibited HCV-induced DR6 expressions at mRNA (Fig. 2B) and promoter activity (Fig. 2C) levels in a dose-dependent manner. To further confirm these results, we quantified the nuclear and cytoplasmic fractions of NF-κB. As shown in Fig. 2D, nuclear fraction of NF-κB was increased by HCV infection (lane 6). However, nuclear translocation of NF-κB was decreased by SN50 in a dose-dependent manner (Fig. 2D, lanes 7 and 8). Collectively, these data indicate that HCV upregulates DR6 expression through NF-κB pathway. Since HCV modulates NF-κB activity via Ca^2+^ signaling and ROS production^13^, we assessed whether Ca^2+^ or ROS was involved in upregulation of DR6 in HCV infected cells. For this purpose, Huh7.5 cells infected with Jc1 were treated with an antioxidant, N-acetylcysteine (NAC). As shown in Fig. 2E, both mRNA (left panel) and protein (right panel) levels of DR6 were markedly decreased by NAC in a dose-dependent manner. On the contrary, intracellular Ca^2+^ chelator (BAPTA-AM) displayed no effect on mRNA (Fig. 2F, left panel) and protein (Fig. 2F, right panel) levels of DR6. This showed that ROS, but not Ca^2+^, was involved in DR6 expression. We further showed that nuclear NF-κB levels in Jc1-infected cells were decreased by NAC in a dose-dependent manner (Supplementary Fig. S3). All these data indicate that HCV upregulates DR6 via ROS-mediated NF-κB pathway.

### DR6 is mainly upregulated by NS5A

**Figure 3:**
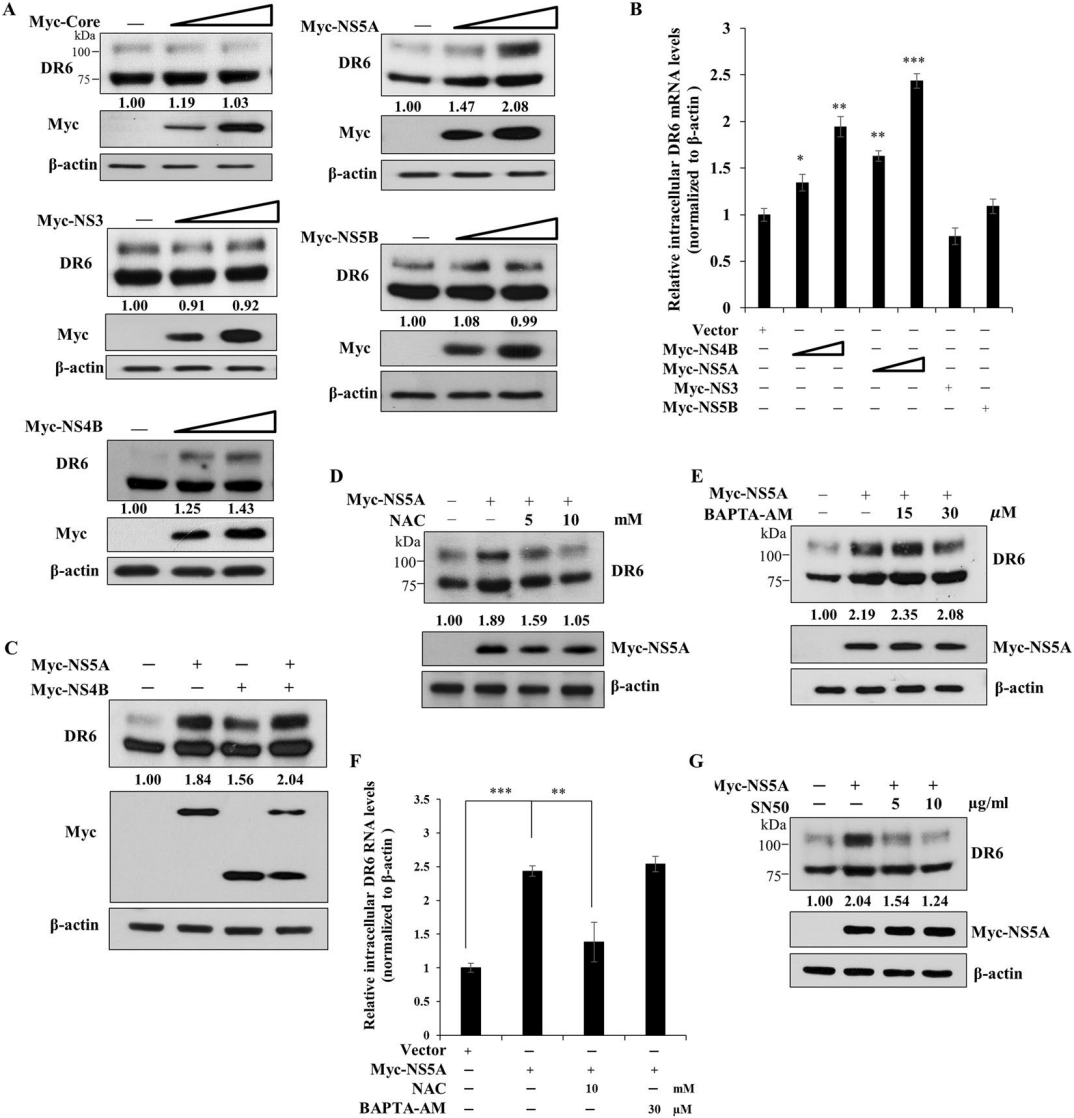
DR6 expression level is mainly upregulated by NS5A protein. (**A**) Huh7.5 cells were transiently transfected with either vector or increasing amounts of Myc-tagged Core, NS3, NS4B, NS5A, or NS5B expression plasmid, individually. At 48 h after transfection, immunoblot analysis was performed to analyze DR6 protein levels. **(B**) Huh7.5 cells were transiently transfected with vector or increasing amount of Myc-tagged NS4B or NS5A expression plasmid. At 48 h after transfection, mRNA level of DR6 was determined by qRT-PCR. Experiments were performed in triplicate. The asterisks indicate significant differences (*P < 0.05, **P < 0.01, ***P < 0.001) from the value for the control. (**C**) Huh7.5 cells were cotransfected with Myc-tagged NS4B and NS5A expression plasmids. At 48 h after transfection, immunoblot analysis was performed to determine DR6 protein levels. Protein band intensities were analyzed by using ImageJ. (**D**) Huh7.5 cells were transfected with either vector or Myc-tagged NS5A. At 48 h after transfection, cells were either left untreated or treated with increasing amounts of NAC. At 48 h after treatment, total cell lysates were immunoblotted with the indicated antibodies. (**E**) Huh7.5 cells were transfected with either vector or Myc-tagged NS5A. At 48 h after transfection, cells were either left untreated or treated with increasing amounts of BAPTA-AM. At 48 h after treatment, total cell lysates were immunoblotted with the indicated antibodies. (**F**) Huh7.5 cells were transfected with either vector or Myc-tagged NS5A. At 48 h after transfection, cells were either left untreated or treated with increasing amounts of NAC or BAPTA-AM. At 48 h after treatment, intracellular mRNA levels of DR6 were analyzed by qRT-PCR. Experiments were performed in triplicate. (**G**) Huh7.5 cells were transfected with either vector or Myc-tagged NS5A. At 48 h after transfection, cells were either left untreated or treated with increasing amounts of SN50. At 48 h after treatment, total cell lysates were immunoblotted with the indicated antibodies.

Since DR6 level was increased not only in HCV-infected cells but also in HCV replicon cells, we hypothesized that viral nonstructural proteins might be responsible for this upregulation. For this purpose, Huh7.5 cells were transiently transfected with either an empty vector or Myc-tagged core, NS3, NS4B, NS5A, NS5B, individually and then protein levels of DR6 were analyzed. As shown in Fig. 3A, DR6 protein levels were increased by either NS4B or NS5A. However, core and other nonstructural proteins displayed no effect on DR6 protein expression. Consistently, transcriptional levels of DR6 were also augmented by either NS4B or NS5A in a dose-dependent manner (Fig. 3B). Of note, DR6 expression level was increased more strongly by NS5A than NS4B. We demonstrated that both NS4B and NS5A displayed an additive effect on protein expression levels of DR6 (Fig. 3C). Since DR6 expression was remarkably upregulated by NS5A, we further explored the functional involvement of NS5A in DR6 upregulation. For this purpose, Huh7.5 cells were transfected with Myc-tagged NS5A and then further treated with increasing amounts of either NAC or BAPTA-AM. Total cell lysates harvested at 48 h after transfection were immunoblotted with the indicated antibodies as shown on the figures. We demonstrated that NS5A-induced DR6 protein levels were decreased only by NAC (Fig. 3D) but not by BAPTA-AM (Fig. 3E). Consistently, NS5A-induced DR6 mRNA levels were decreased by NAC but not by BAPTA-AM (Fig. 3F). We observed the similar results by using Myc-tagged NS4B (Supplementary Fig. S4). We further verified that NS5A-induced DR6 expression levels were decreased by SN50 (Fig. 3G). These data indicate that NS5A is crucially involved in upregulation of DR6 and this upregulation occurs through ROS-induced NF-κB activation in HCV-infected cells.

### HCV NS5A specifically interacts with DR6

**Figure 4:**
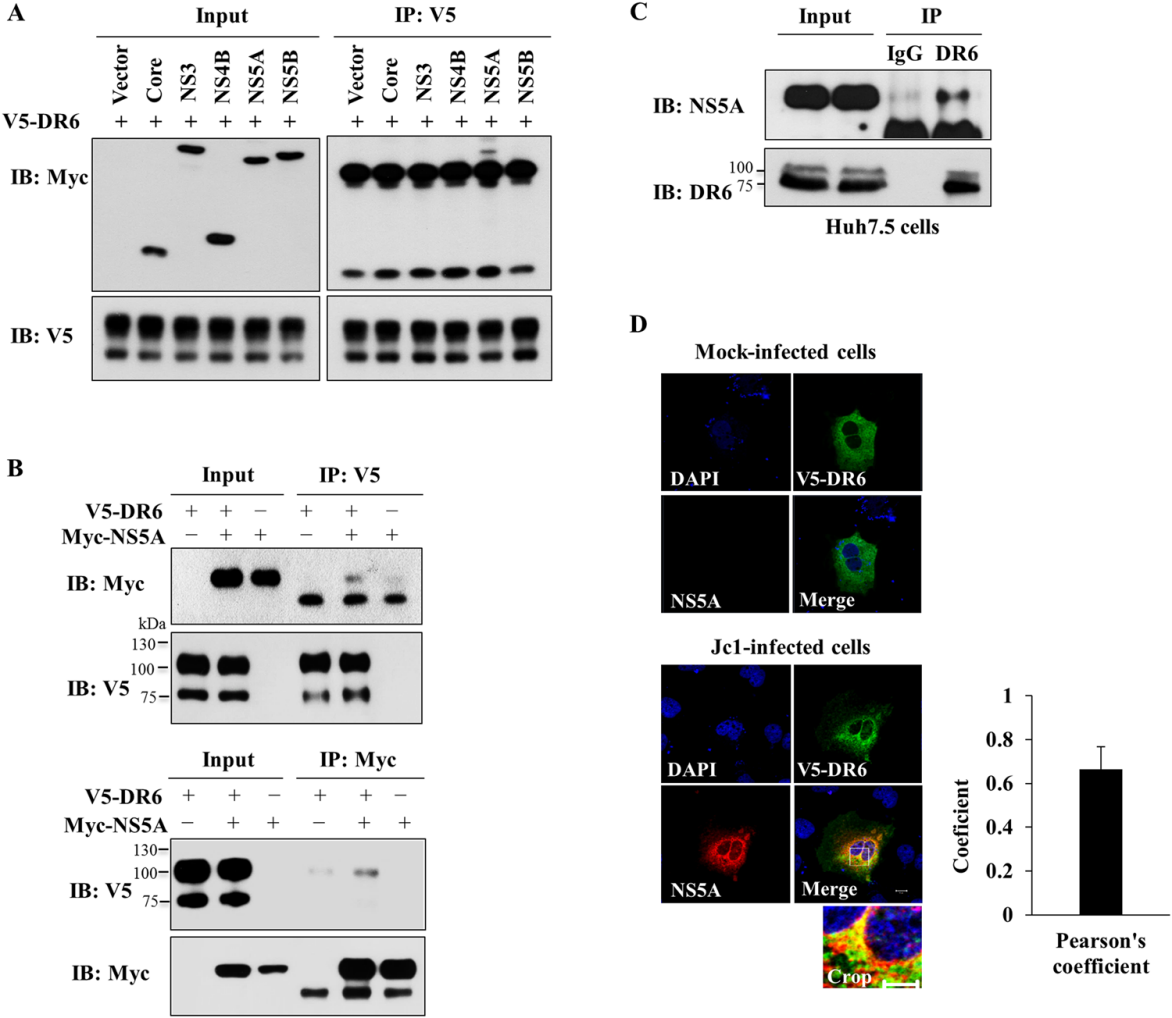
DR6 interacts with NS5A protein. (**A**) Huh7.5 cells were cotransfected with V5-tagged DR6 and vector or the indicated each of Myc-tagged HCV protein expression plasmid, respectively. At 48 h after transfection, cell lysates were immunoprecipitated with an anti-V5 antibody and then bound proteins were detected by immunoblot analysis using an anti-Myc antibody. (**B**) (Upper panel) Huh7.5 cells were cotransfected with V5-tagged DR6 and Myc-tagged NS5A expression plasmids. At 48 h after transfection, cell lysates were immunoprecipitated with an anti-V5 monoclonal antibody and then bound proteins were detected by immunoblot assay using an anti-Myc monoclonal antibody. Protein expression of Myc-tagged NS5A and V5-tagged DR6 were verified by immunoblotting with the indicated antibody. (Lower panel) Cell lysates were immunoprecipitated with an anti-Myc monoclonal antibody and bound proteins were detected by immunoblot assay using an anti-V5 monoclonal antibody. **(C**) Huh7.5 cells were electroporated with *in vitro*-transcribed Jc1 RNA. Four days after electroporation, total cell lysates were immunoprecipitated with either mouse IgG or mouse anti-DR6 antibody. Bound proteins were immunoblotted with the indicated antibodies. (**D**) Huh7 cells were either mock-infected or infected with Jc1. At 48 h postinfection, cells were further transfected with V5-tagged DR6 expression plasmid. At 24 h after transfection, cells were fixed in 4% paraformaldehyde and immunofluorescence staining was performed by using an anti-V5 monoclonal antibody and fluorescein isothiocyanate-conjugated goat anti-mouse IgG to detect V5-tagged DR6 (green), and rabbit anti-NS5A antibody and TRITC-conjugated donkey anti-rabbit IgG to detect NS5A (red). Dual staining showed colocalization of DR6 and NS5A as yellow fluorescence in the merged image. Cells were counterstained with DAPI to label nuclei (blue). Scale bar = 5 μm. The enlarged section marked by a white square is shown as a crop image. Colocalization of DR6 and NS5A was verifed by Pearson’s overlap coefficient. More than 10 cells were applied to ImageJ for quantification of overlap coefficient, and error bar indicates the standard deviation of the mean.

Growing evidence shows that HCV proteins bind to host cellular proteins and modulate cellular properties and function^14, 15^. We therefore explored the possible protein interaction between DR6 and HCV proteins. For this purpose, Huh7.5 cells were cotransfected with V5-tagged DR6 and each of Myc-tagged viral proteins expression plasmid, followed by an immunoprecipitation assay. As shown in Fig. 4A, only NS5A interacted with DR6. We further verified that V5-tagged DR6 specifically interacted with Myc-tagged NS5A (Fig. 4B, upper panel). By reciprocal experiment, Huh7.5 cells cotransfected with V5-tagged DR6 and Myc-tagged NS5A plasmids were immunoprecipitated with an anti-Myc antibody, and bound proteins were analyzed by immunoblotting with an anti-V5 antibody. We confirmed that NS5A specifically interacted with DR6 (Fig. 4B, lower panel). Finally, we demonstrated that endogenous DR6 specifically interacted with NS5A in HCV-infected Huh7.5 cells (Fig. 4C). The interaction between DR6 and NS5A suggests that NS5A might colocalize with DR6. To investigate this possibility, Huh7 cells were either mock infected or infected with Jc1 and then further transfected with V5-tagged DR6 expression plasmid. At 24 h after transfection, a confocal immunofluorescence assay was performed. As shown in Fig. 4D, both NS5A and DR6 were colocalized in the cytoplasm in Jc1-infected cells as indicated by yellow fluorescence in the merged image. Collectively, these data indicate that NS5A specifically interacts with DR6.

### HCV modulates JNK, p38 MAPK, STAT3, and Akt signaling pathway in a DR6-dependent manner

**Figure 5:**
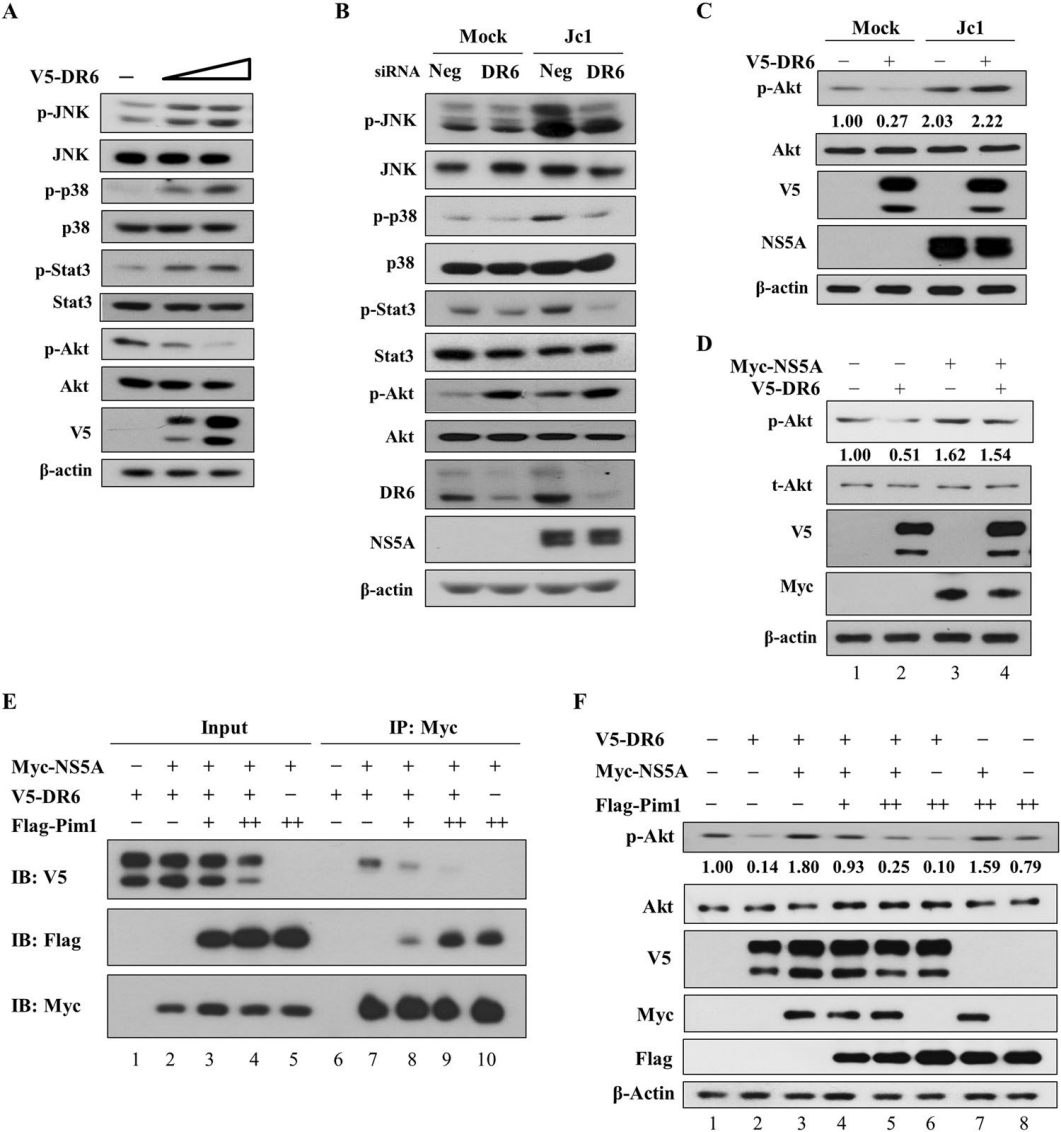
HCV regulates JNK, p38 MAPK, STAT3, and Akt activities through DR6. (**A**) Huh7.5 cells were transfected with either empty vector or increasing amounts of V5-tagged DR6 expression plasmid. At 48 h after transfection, protein levels were analyzed by immunoblot analysis using the indicated antibodies. (**B**) Huh7.5 cells were either mock-infected or infected with Jc1 for 4 h. At 48 h postinfection, cells were further transfected with 20 nM of the negative or DR6 siRNAs. At 48 h after transfection, protein levels were analyzed by immunoblot assay using the indicated antibodies. Neg denotes universal negative control siRNA. (**C**) Huh7.5 cells were either mock-infected or infected with Jc1 for 4 h. At 48 h postinfection, cells were further transfected with V5-tagged DR6 expression plasmid. At 48 h after transfection, protein levels were analyzed by immunoblot analysis using the indicated antibodies. (**D**) Huh7.5 cells were cotransfected with V5-tagged DR6 and Myc-tagged NS5A expression plasmid. At 48 h after transfection, total cell lysates were immunoblotted with the indicated antibodies. Protein band intensities of p-Akt/Akt were analyzed by using ImageJ. **(E**) Huh7.5 cells were cotransfected with Myc-tagged NS5A, V5-tagged DR6, and Flag-tagged Pim1 expression plasmids. At 48 h after transfection, total cell lysates were immunoprecipitated with an anti-Myc antibody and then bound proteins were detected by immunoblot analysis using an anti-V5 and an anti-Flag antibody, respectively. (**F**) Huh7.5 cells were cotransfected with V5-tagged DR6, Myc-tagged HCV NS5A, and Flag-tagged Pim1 expression plasmids. At 48 h after transfection, total cell lysates were immunoblotted with the indicated antibodies. Protein band intensities of p-Akt/Akt were analyzed by using ImageJ.

It has been previously reported that DR6 is involved in tumor angiogenesis via NF-kB, p38 MAPK and STAT3 pathways^9^. Since HCV infection modulates JNK, p38 MAPK, STAT3, and Akt signaling pathways^16–19^, we investigated whether DR6 was involved in these HCV-mediated signaling pathways. For this purpose, V5-tagged DR6 was ectopically expressed in Huh7.5 cells and the signaling cascades were analyzed by immunoblot analysis using the indicated antibodies. As shown in Fig. 5A, phosphorylation levels of JNK, p38 MAPK and STAT3 were increased by DR6 in a dose-dependent manner, whereas phosphorylation level of Akt was decreased by overexpression of DR6 protein. To further verify these results in the context of HCV replication, Huh7.5 cells infected with Jc1 were further transfected with a DR6-specific siRNA. As shown in Fig. 5B, HCV infection increased phosphorylation levels of JNK, p38 MAPK, STAT3, and Akt (Fig. 5B). Knockdown of DR6 in HCV-infected cells impaired HCV-induced phosphorylation of JNK, p38 MAPK and STAT3 as compared with those in mock-infected cells. Surprisingly, silencing of DR6 promoted the phosphorylation level of Akt in HCV-infected cells. To further investigate the effect of DR6 on phosphorylation level of Akt in HCV-infected cells, Huh7.5 cells were either mock-infected or infected with Jc1 and then further transfected with V5-tagged DR6 expression plasmid. As shown in Fig. 5C, overexpression of DR6 suppressed the phosphorylation of Akt in mock-infected cells. However, overexpression of DR6 in Jc1-infected cells no longer suppressed the phosphorylation of Akt. Neither overexpression nor silencing of DR6 displayed effect on cell growth (Supplementary Fig. S5). Since DR6 interacted with NS5A, this suggested that NS5A might interrupt DR6 function via protein interplay. To corroborate this result, Huh7.5 cells were cotransfected with V5-tagged DR6 and Myc-tagged NS5A expression plasmid and then phosphorylation level of Akt was determined. As shown in Fig. 5D, inhibitory activity of DR6 on Akt phosphorylation was disrupted by NS5A (lane 2 versus lane 4). These data demonstrated that NS5A nullified the inhibitory function of DR6 on Akt phosphorylation in HCV-infected cells. To further verify whether NS5A impaired DR6 function by protein interplay, we took advantage of using Pim1, a member of serine/threonine kinase, as a competitor for NS5A binding. We previously reported that Pim1 interacts with HCV NS5A and regulates HCV entry^15^. For this purpose, Huh7.5 cells were cotransfected with Myc-tagged NS5A and V5-tagged DR6 in the absence or presence of Flag-tagged Pim1 plasmid. Figure 5E shows that DR6 specifically interacted with NS5A (lane 7). Of note, protein interaction between DR6 and NS5A was reduced by Pim1 in a dose-dependent manner. To investigate if protein interaction between DR6 and NS5A dispayed a negative effect on DR6 activity in Akt phosphorylation, Huh7.5 cells were cotransfected with V5-tagged DR6 and Myc-tagged HCV NS5A plasmids in the absence or presence of Flag-tagged Pim1 plasmid. At 48 h after transfection, phosphorylation level of Akt was analyzed. As shown in Fig. 5F, ectopic expression of Pim1 displayed no discernable effect on Akt phosphorylation compared with vector transfected cells (lane 8 versus lane 1). As expected, inhibitory activity of DR6 on Akt phosphorylation was impaired by NS5A (Fig. 5F, lane 3 versus lane 2). Importantly, inhibitory activity of DR6 on Akt phosphorylation was gradually rescued by Pim1 in a dose-dependent manner (lanes 4 and 5). Collectively, these data indicate that NS5A disrupts DR6-mediated Akt signaling in HCV-infected cells.

### DR6 is required for HCV propagation

**Figure 6:**
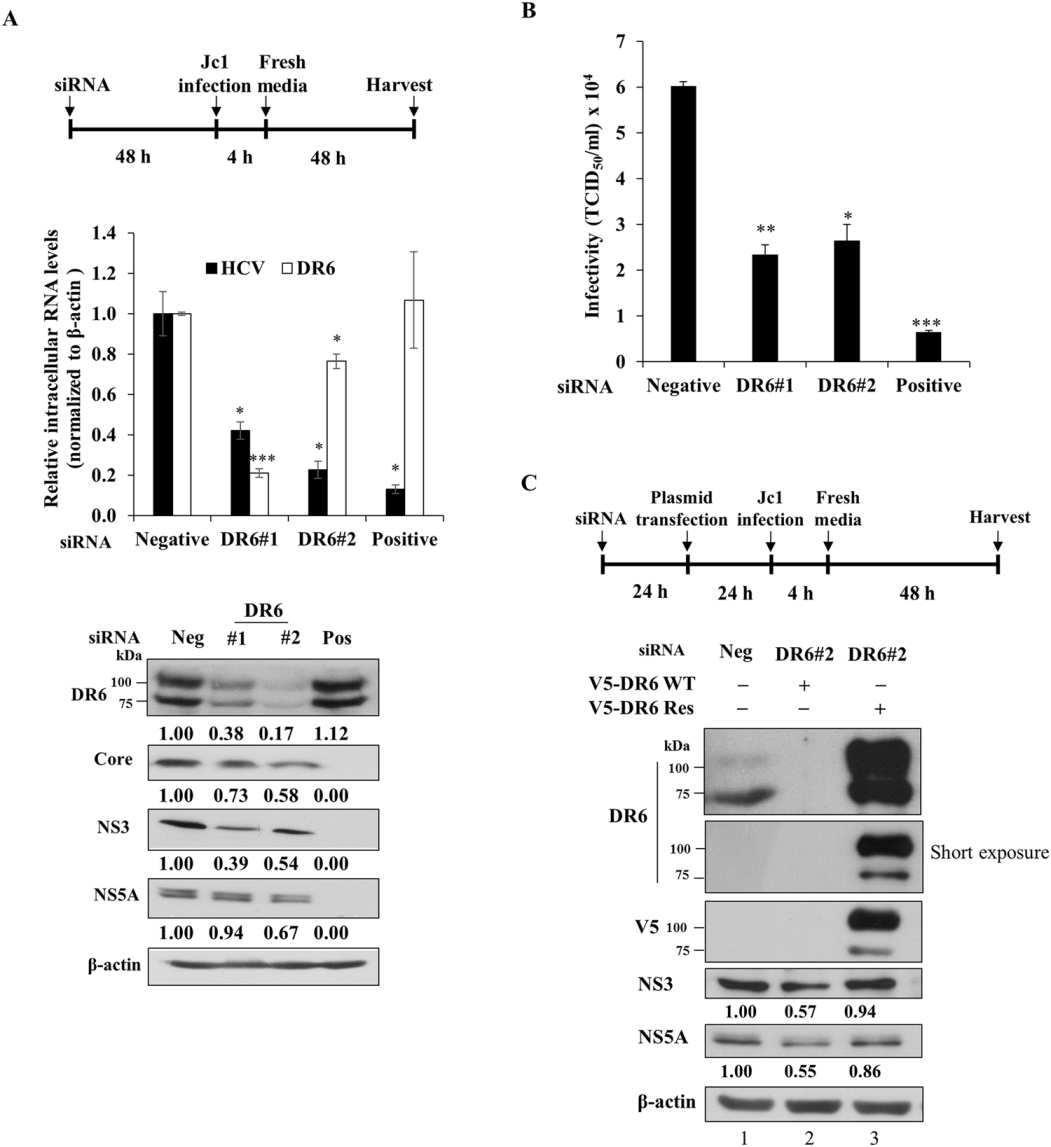
DR6 is required for HCV propagation. (**A**) (Top panel) Schematic illustration of the experimental design. Huh7.5 cells were transfected with 20 nM of the indicated siRNAs. At 2 days after siRNA transfection, cells were infected with Jc1. At 48 h postinfection, both RNA (middle panel) and protein levels (bottom panel) were analyzed by qRT-PCR and immunoblot assay using the indicated antibodies, respectively. The asterisks indicate significant differences (*P < 0.05; **P < 0.01; ***P < 0.001) from the value for the negative control. Neg, negative universal control siRNA; Pos, positive HCV-specific siRNA targeting the 5′ nontranslated region (NTR) of Jc1. (**B**) Huh7.5 cells were transfected with 20 nM of the indicated siRNAs. At 2 days after transfection, cells were infected with green fluorescent protein-tagged Jc1. At 48 h postinfection, HCV infectivity was determined by limiting dilution assay. Infected cells were assessed by fluorescent microscope. TCID_50_, tissue culture infectious dose 50%. (**C**) (Upper panel) Schematic illustration of the experimental design. (Lower panel) Huh7 cells were transfected with the indicated siRNAs. At 24 h after siRNA transfection, cells were further transfected with either wild-type or siRNA-resistant DR6 mutant plasmid and followed by Jc1 infection. At 48 h postinfection, cell lysates were immunoblotted using the indicated antibodies. Res, V5-tagged siRNA-resistant mutant of DR6. Protein band intensities were analyzed by using ImageJ. All experiments were performed in triplicate.

To investigate whether DR6 is involved in HCV propagation, Huh7.5 cells were transfected with two different siRNAs targeting DR6 first and then infected with Jc1 as depicted in the schematic illustration in Fig. 6A (top panel). At 48 h postinfection, both RNA and protein levels were analyzed. Silencing of DR6 significantly suppressed HCV RNA levels (Fig. 6A, middle panel). Consistently, HCV protein expressions were impaired in DR6-knockdown cells (Fig. 6A, bottom panel). We also determined the 50% tissue culture infectious dose (TCID_50_) of HCV in DR6-knockdown cells. Figure 6B shows that HCV infectivity was significantly decreased in DR6-knockdown cells. To rule out the off-target effect of a DR6 siRNA, we generated a siRNA-resistant DR6 mutant. We showed that exogenous expression of the siRNA-resistant DR6 mutant, but not of wild-type DR6, rescued the HCV protein expression level (Fig. 6C, lane 3 versus lane 2). These data indicate that DR6 is required for HCV propagation.

### DR6 is not involved in the entry, replication, and translation steps of the HCV life cycle

**Figure 7:**
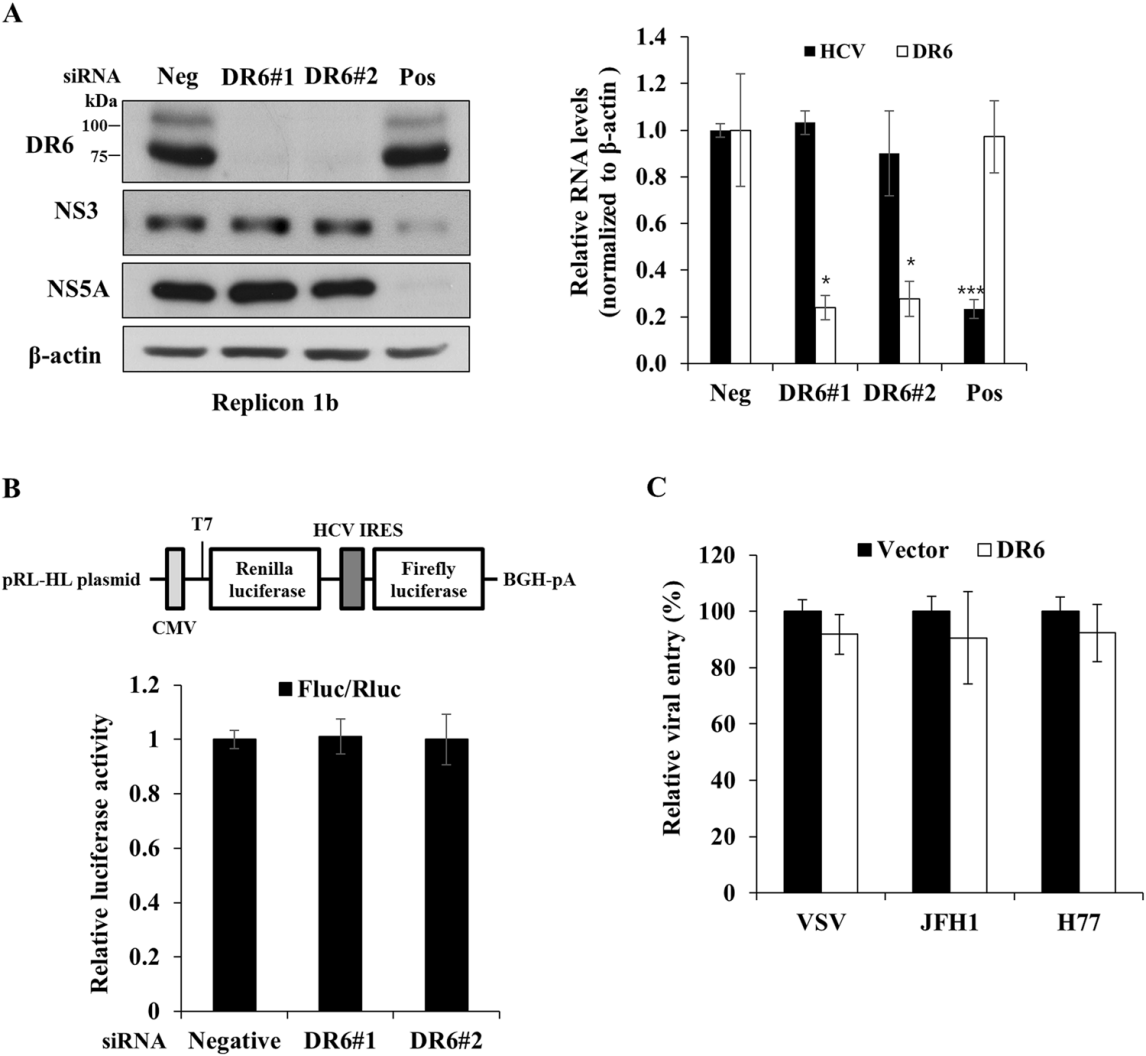
DR6 is not involved in the entry, replication, and translation steps of the HCV life cycle. (**A**) Huh7 cells harboring HCV subgenomic replicon derived from genotype 1b were transfected with the indicated siRNAs. At 72 h after siRNA transfection, both RNA (left panel) and protein (right panel) levels were analyzed by qRT-PCR and immunoblot assays, respectively. (**B**) (Upper panel) Schematic diagram of pRL-HL plasmid. (Lower panel) Huh7.5 cells were transiently transfected with the indicated siRNAs for 48 h and then further transfected with pRL-HL dual luciferase and pCH110 β-galactosidase plasmid. At 48 h after transfection, relative luciferase activities were determined. (**C**) Huh7.5 cells were transfected with either vector or V5-tagged DR6 expression plasmid for 48 h and then infected with either VSVpp or HCVpp derived from genotype 1a (H77) or 2a (JFH1) for 6 h. At 72 h postinfection, viral entry was determined by luciferase activity. All experiments were performed in triplicate.

To further dissect which step of viral propagation depended on DR6, Huh7 cells harboring HCV subgenomic replicon derived from genotype 1b were transfected with DR6-specific siRNA and then both HCV RNA and protein levels were analyzed. As shown in Fig. 7A, silencing of DR6 exerted no effect on HCV RNA (left panel) and protein (right panel) levels in HCV replicon cells. We then analyzed whether DR6 was involved in HCV internal ribosome entry site (IRES)-mediated translation. For this purpose, Huh7.5 cells transfected with siRNAs were further transfected with pRL-HL plasmid (Fig. 7B, upper panel) and then relative luciferase activities were determined. We showed that knockdown of DR6 displayed no effects on HCV IRES-dependent translation (Fig. 7B, lower panel). We then asked if DR6 was involved in HCV entry step of the HCV life cycle. Huh7.5 cells were transfected with either an empty vector or V5-tagged DR6 expression plasmid and then infected with either VSVpp or HCVpp derived from genotype 1a (H77) or 2a (JFH1) as we reported previously^15^. Figure 7C shows that overexpression of DR6 displayed no discernable effects on HCV entry. These data suggest that DR6 might be involved in other steps of the HCV life cycle.

### DR6 is involved in the production of infectious virus

**Figure 8:**
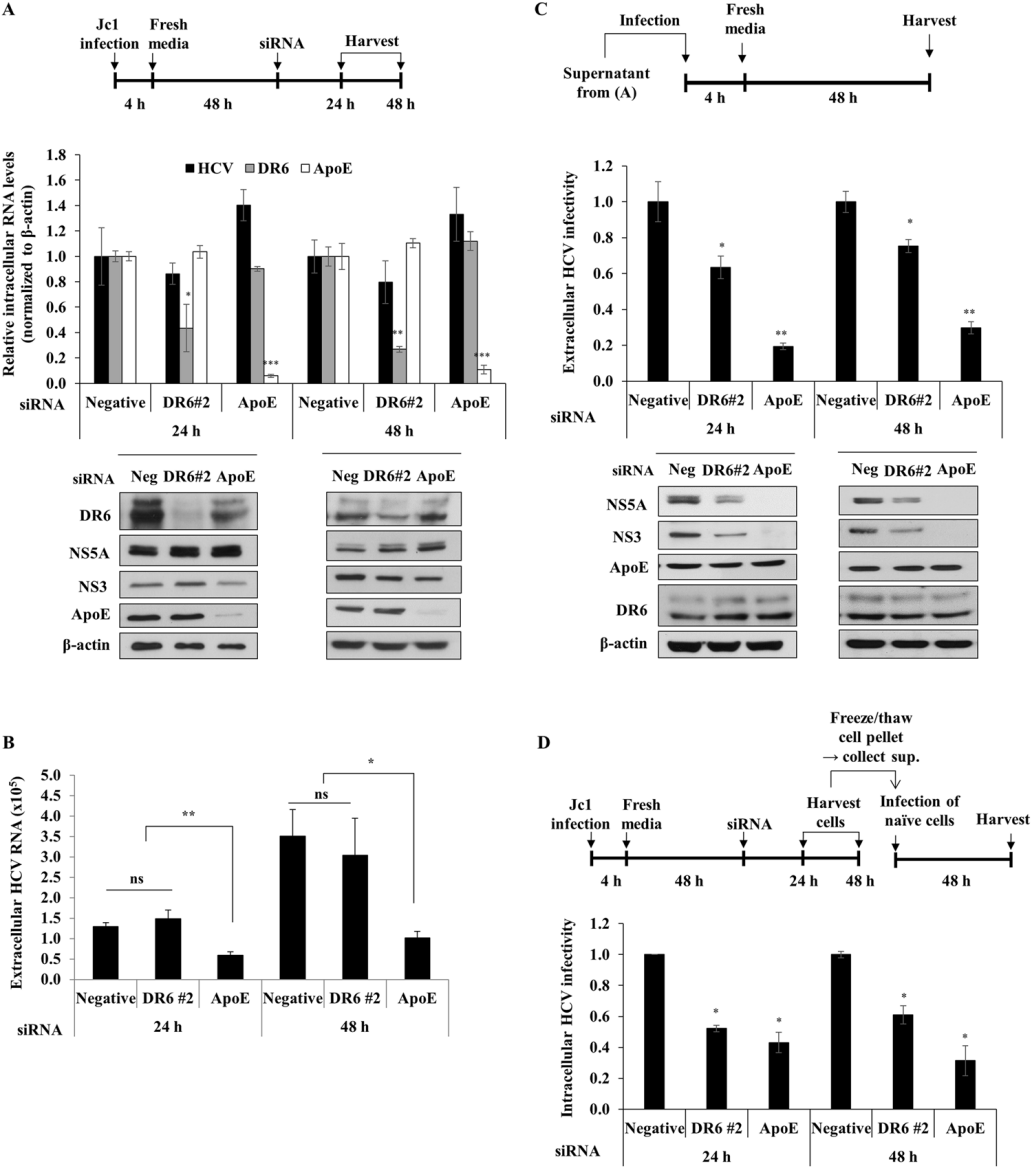
DR6 is required for a late step of the HCV life cycle. (**A**) (Top panel) Schematic illustration of the experimental design. Huh7.5 cells were infected with Jc1 for 4 h. At 48 h postinfection, cells were transfected with 20 nM of the indicated siRNAs. At the indicated time points, both intracellular RNA (middle panel) and protein (bottom panel) were determined by qRT-PCR and immunoblot assays, respectively. siRNA targeting ApoE was used as a positive control for HCV assembly. (**B**) Copy numbers of extracellular HCV RNA in supernatant collected from (**A**) were analyzed by qRT-PCR. (**C**) Naïve Huh7.5 cells were infected with Jc1 harvested from culture supernatant of panel A. At the indicated time points after infection, both extracellular HCV infectivity (upper panel) and protein (lower panel) levels were determined by qRT-PCR and immunoblot assays, respectively. (**D**) (Upper panel) Schematic illustration of the experimental design. (Lower panel) Huh7.5 cells treated as described in (**A**) were lysed with three freeze-thaw cycles and centrifuged at 15,000 × g for 15 min. The cellular supernatant was collected and used to infect naïve Huh7.5 cells. At 48 h postinfection, relative intracellular HCV infectivity was determined by qRT-PCR. Negative indicates universal control siRNA. All experiments were performed in triplicate.

We next asked whether DR6 was required for the later stages of the HCV life cycle. Huh7.5 cells were infected with Jc1 first and then transfected with siRNAs as depicted in the schematic illustration in Fig. 8A (top panel). We used apolipoprotein E (ApoE) siRNA as a control. In spite of significant suppression of DR6 mRNA level, knockdown of DR6 showed no effect on intracellular HCV RNA levels (Fig. 8A, middle panel). Consistently, knockdown of DR6 displayed no effect on protein levels of HCV (Fig. 8A, bottom panel). We further demonstrated that extracellular HCV RNA copy numbers were not altered in DR6-knockdown cells (Fig. 8B). However, when naïve Huh7.5 cells were infected with Jc1 harvested from the culture supernatant of the experiment shown in Fig. 8A, intracellular HCV RNA levels were significantly decreased in cells infected with Jc1 from culture supernatant of DR6-enervated cells (Fig. 8C, upper panel). Consistently, HCV protein levels were markedly reduced in cells infected with Jc1 from culture supernatant of DR6-enervated cells (Fig. 8C, lower panel). These data implied that DR6 might be involved in production of infectious virus. To further verify these results, intracellular HCV infectivity was determined. Huh7.5 cells were infected with Jc1 first and then transfected with the indicated siRNAs as depicted in the schematic illustration in Fig. 8D (upper panel). Cells harvested either at 24 h or 48 h postinfection were lysed by repeated cycles of freezing and thawing and then intracellular HCV were isolated. As shown in Fig. 8D (lower panel), infection of naïve Huh7.5 cells with HCV purified from the cells resulted in significant decrease of intracellular HCV infectivity in DR6-knockdown cells. Since knockdown of DR6 impaired HCV infectivity (Fig. 8C and D) but not intracellular and extracellular HCV RNA expression levels (Fig. 8A and B), these data indicate that DR6 is involved in production of infectious virus.

## Discussion

Our previous transcriptome analysis data showed that DR6 was highly expressed in HCVcc-infected cells^12^. DR6, a member of tumor necrosis factor related death receptor family, is highly expressed in several prostate tumor cell lines^10^. DR6 is considered as a potential biomarker in several adult sarcomas^20^. It has been reported that mRNA levels of DR6 in rat hepatocytes were dramatically increased during liver regeneration^21^. mRNA level of DR6 was also moderately upregulated in peripheral blood mononuclear cells of chronic HCV patients^22^. However, there has been no evidence that DR6 is involved in virus-mediated liver diseases. In the present study, we demonstrated that both mRNA and protein levels of DR6 were upregulated by HCV. We also showed that DR6 promoter activity was upregulated by HCV. DR6 is upregulated by TNF-α, which has ability to elevate NF-κB and its target gene interleukin-6 (IL-6)^23^. We showed that overexpression of NS5A promoted DR6 expression level. In fact, NS5A is a multifunctional protein that can induce oxidative stress and activate STAT-3 and NF-κB^24^. In addition, we observed that domain I (1–249 aa) of NS5A was responsible for the upregulation of DR6 expression (data not shown), which is consistent with the fact that domain I of NS5A triggers ROS production^13^. We further verified that HCV-induced DR6 upregulation was impaired by SN50, confirming that HCV upregulates DR6 expression via NF-κB pathway. Collectively, these data suggest that HCV upregulates DR6 expression through oxidative stress-mediated NF-κB activation.

We also demonstrated that overexpression of NS4B increased DR6 expression level. Although the effect of NS4B on DR6 expression was lower than that of NS5A, it was not surprising because NS4B has ability to stimulate ROS production in hepatocytes^25^. While NS5A increased both 75 kDa and 110 kDa protein expression levels, NS4B mainly upregulated 110 kDa protein expression. The underlying mechanism for the viral protein-specific upregulation of DR6 is largely unknown. DR6 is expressed as multiple protein bands due to its different glycosylated forms^7^. Indeed, we confirmed that O-glycosylation was responsible for the larger molecular weight of DR6 (data not shown). Multiple potential O-linked oligosacharide chain sites are required for sorting and leading to effective transportation of DR6^7^. It has been previously reported that O-glycosylation of DR4 and DR5 promotes ligand-induced receptor clustering and activation of apototic signaling in cancer cell lines^26^. Nevertheless, the implication of O-glycosylation in DR6 has not been functionally demonstrated as yet. Thus how glycosylation of DR6 is involved in the production of infectious HCV will be an interesting aspect of future study.

DR6 has been shown to be involved in JNK, p38 MAPK and STAT3 signaling^6, 9^ and HCV infection modulates JNK, p38 MAPK, STAT3, and Akt signaling pathways^24, 27^. Indeed, we showed that DR6 is required for the phosphorylation of JNK, p38 MAPK and STAT3 in HCV-infected cells. STAT3 activation is linked to the growth of human hepatocellular carcinoma both *in vitro* and *in vivo*, including proliferation, survival, and angiogenesis^27^. HCV also enhances TGF-β1 production via JNK and p38 MAPK-dependent pathway^28^, which induces hepatic fibrogenesis in HCV infected cells^29^. The regulatory function of DR6 in JNK, p38 MAPK, and STAT3 pathways suggests that DR6 may mediate pathogenesis in HCV-infected cells. The C-terminal death domain is responsible for pro-apoptotic function of DR6 in several cell lines^6, 8^. Although there is no direct evidence showing pro-apoptotic role of DR6 in hepatocytes, the study of liver regeneration in rats suggests that DR6 may play a role in hepatocyte apoptosis^21^. Blocking DR6 function promotes human motor neuron survival through activation of Akt phosphorylation and inhibition of caspase 3^30^. We also showed that silencing of DR6 increased Akt activity in hepatocytes. We further demonstrated that overexpression of DR6 suppressed the phosphorylation of Akt in mock-infected cells. However, overexpression of DR6 no longer suppressed the phosphorylation of Akt in HCV-infected cells. Although HCV upregulated DR6 expression level, DR6 was unable to suppress Akt activity in HCV-infected cells. Since DR6 interacted with NS5A, we speculated that NS5A might impair DR6-mediated Akt activation. It has been previously reported that HCV activates N-Ras-PI3K-Akt-mTOR pathway to promote cell survival^17^ and thus blocking Akt-inhibiotry function of DR6 may assist normal Akt activation process. Since DR6-mediated Akt activity is impaired in HCV-infected cells, these data suggest that HCV coopts DR6 to regulate Akt signaling cascade to favor viral propagation and it may also contribute to liver pathogenesis.

Death receptors and ligands play roles in virus-induced apoptosis without stimulating viral propagation^31^. For example, HCV-induced TNF-α activates TNFR1 to regulate viral replication^32^. However, in the present study, DR6 functions as a proviral factor for the HCV propagation. Silencing of DR6 impaired both HCV RNA and protein levels. HCV infectivity was also significantly reduced by knockdown of DR6 without causing cellular toxicity. We showed that DR6 was not involved in the entry, RNA replication, and IRES-mediated translation steps of the HCV life cycle. However, knockdown of DR6 significantly reduced the intracellular HCV infectivity. These data suggest that DR6 is involved in the production of infectious HCV. HCV virion production can be divided into several steps, including assembly, maturation, and secretion^33^. Which stage of the HCV life cycle specifically requires DR6 remains to be determined. N-glycosylation of DR6 is responsible for lipid-raft localization^7^. Here, we showed that DR6 colocalized with NS5A and hence it might be possible to participate in replication complex. This is reminiscent of ANXA2 which is recruited to the viral replication sites and involved in viral assembly^34, 35^. Since NS5A plays a central role in the recruitment of the replication complex to assembly sites for genome encapsidation and nucelocapsid formation^36, 37^, DR6 may be recruited to the replication sites by NS5A and participates in viral production. Interestingly, it has been recently reported that HCV release occurs via a trans-Golgi network (TGN) to endosome pathway that is distinct from that of lipid droplet-associated endoplasmic reticulum^38^. Since other death receptors, TNFR1^39, 40^, TNFR2^41^, DR4, and DR5^42^, show the TGN localization in several cell lines, it would be interesting to investigate if DR6 plays any role in TGN-associated HCV secretion.

DR6 is the least studied receptor in death receptor family. Because the ligand of DR6 is unknown, the molecular signaling mechanisms of DR6 have not been demonstrated yet. Here, we demonstrate that HCV upregulates DR6 expression level via ROS-mediated NF-κB pathway. Furthermore, HCV exploits DR6 signaling pathway to facilitate production of infectious virus. Since DR6 is not only involved in HCV propagation but also engaged in HCV-mediated JNK, p38 MAPK, STAT3, and Akt signaling pathways, it may also contribute to HCV-associated pathogenesis.

## Methods

### Plasmid constructions and DNA transfection

Total cellular RNAs were extracted from Huh7 cells by using RiobEx (GeneAll), and cDNA was synthesized by using a cDNA synthesis kit (Toyobo) according to the manufacturer’s instructions. Full-length DR6 was amplified by using primer sets listed in Table 1. PCR product was inserted into the *Not*I and *Xba*I sites of plasmid pEF6/V5-HisB (Invitrogen). A small interfering RNA (siRNA)-resistant DR6 mutant (pEF6/V5-DR6-Res) was constructed by introducing three mutations at the siRNA-binding site using PCR-based site-directed mutagenesis with primers listed in Table 1. All DNA transfections were performed by using a polyethyleneimine reagent (Sigma-Andrich) as we described previously^43^.

**Table 1:** List of primers used in this study.

Primer	Primer sequence	Purpose
DR6-F (*Not*I)	AAGCGGCCGCAATGGGGACCTCTCC	Cloning of human DR6 into pEF6/V5-HisB vector
DR6-R (*Xba*I)	GCTCTAGAACCAACAGCAGGTCAGGAAGA	
DR6 resist-mutant-F	AGCAGGAACGGTTCGTTCATCACCAAAGAAAAG	Generation of siRNA-resistant mutant of DR6
DR6 resist-mutant-R	CTTTTCTTTGGTGATGAACGAACCGTTCCTGCT	
pDR6-F	GCTAGCATAATCTATCACTCTATAGAGGGA	Cloning of DR6 promoter into pGL3-basic vector
pDR6-R	AAGCTTACTGAGTCGGTGGCCA	
pDR6- NF-κB-mut1-F	TCCTTTTATTATGAAGGTTTTAATTGCATT CA	Generation of NF-κB binding site deletion from −1035 to −1023 of DR6 promoter
pDR6- NF-κB-mut1-R	TGAATGCAATTAAAACCTTCATAATAAAAGGA	
pDR6- NF-κB-mut2-F	TGAACTCTCCAATTGAGGATACAGGGCAGC	Generation of NF-κB binding site deletion from −817 to −805 of DR6 promoter
pDR6- NF-κB-mut2-R	CGCTGCCCTGTATCCTCAATTGGAGAGTTC	
5′NTR-F	TGAGTGTCGTACAGCCTCCA	Quantitative real-time PCR
5′NTR-R	ACGCTACTCGGCTAGCAGTC	
qActin-F	TGACAGCAGTCGGTTGGAGCG	Quantitative real-time PCR
qActin-R	GACTTCCTGTAACAACGCATCTCATA	
qDR6-F	TCTTCGTGGATGAGTCGGAG	Quantitative real-time PCR
qDR6-R	GCAAGTCACAGGGGTCCAG	

### Construction of DR6 promoter

DR6 promoter was constructed by using the specific primers listed in Table 1 and by genomic DNA isolated from Huh7 cells. The amplified PCR products were cloned into pGL3-basic vector (Promega) to generate luciferase reporter construct of DR6 promoter. Two putative NF-kB binding sites in DR6 promoter were mutated based on prediction of Matlnspector analyzer (Genomatix). Deletion mutants of NF-κB binding sites on DR6 promoter were generated by PCR using primers listed in Table 1. Luciferase assays were performed as we described previously^44^.

### Antibodies and chemicals

Antibodies were purchased from the following resources: DR6, calnexin, c-Myc, NF-κB p65, Lamin A/C, JNK, STAT3, and p38 antibodies were from Santa Cruz; phospho JNK (Thr183/Tyr185), phospho-p38 (Thr180/Tyr182), and phospho STAT3 (Tyr 705) antibodies were from Cell Signaling; β-actin antibodies were from Sigma-Aldrich; V5 antibodies were from Invitrogen; HCV core, NS3, NS4B and NS5A antibodies have been described elsewhere^43^. SN50, a NF-κB inhibitor, was purchased from EMD Millipore. N-acetyl-cysteine (NAC), an antioxidant agent, and BAPTA-AM, a calcium chelator, were purchased from Sigma-Aldrich and Enzo, respectively.

### Cell culture

All cell lines were cultured in Dulbecco’s modified Eagle’s medium (DMEM) supplemented with 10% fetal bovine serum and 100 units/ml of penicillin-streptomycin in 5% CO_2_ at 37 °C. Huh7 cells harboring HCV subgenomic replicon derived from genotype 1b and IFN-cured cells were grown as reported previously^43^.

### Immunoblot analysis

Cells were washed twice with phosphate-buffered saline (PBS) and lysed in RIPA buffer (50 mM Tris-HCl [pH 7.5], 1% NP-40, 150 mM NaCl, 1 mM EDTA, 1 mM NaF, 1 mM Na_3_VO_4_, and 1 mM phenylmethylsulfonyl fluoride (PMSF) for 15 min on ice and centrifuged at 12,000 rpm for 10 min at 4 °C. The supernatants were collected and equal amounts of protein were subjected to SDS-PAGE and electrotransferred to a nitrocellulose membrane. The membrane was blocked in Tris-buffered saline (TBS)-Tween (20 mM Tris-HCl [pH 7.6], 150 mM NaCl, and 0.25% Tween 20) containing 5% nonfat dry milk for 1 h and then incubated with the indicated antibodies as shown on the figures. Proteins were detected using an ECL kit (Abfrontier).

### Quantification of RNA

Total RNAs were isolated from cells using RiboEX reagent (GeneAll) according to the manufacturer’s instructions. cDNAs were synthesized by using a cDNA synthesis kit (Toyobo) according to the manufacturer’s instructions. Quantitative real-time PCR (qRT-PCR) experiments were performed using primers listed in Table 1. qRT-PCR experiments were done using the CFX Connect real-time system (Bio-Rad Laboratories, Hercules, CA) under the following conditions: 15 min at 95 °C, followed by 40 cycles of 95 °C for 20 s, 55 °C for 20 s, and 73 °C for 20 s. Seventy one cycles of 10 s, with 0.5 °C temperature increments from 60 °C to 95 °C, were used for the melting curves.

### Immunoprecipitation

Huh7.5 cells were cotransfected with V5-tagged DR6 and Myc-tagged HCV core, NS3, NS4B, NS5A, NS5B, respectively. Total amounts of DNA were adjusted by adding an empty vector. At 48 h after transfection, cells were harvested and immunoprecipitation assay was performed using either an anti-Myc and an anti-V5 antibody as we described previously^43^.

### RNA interference

siRNAs targeting two different regions of DR6 (DR6#1 [5′-AGA CCA AAG GUA CUG AGU A-3′ and DR6#2 [5′-ACG GUU CCU UUA UUA CCA A-3′]) and the universal negative control siRNA were purchased from Bioneer (South Korea). siRNA targeting the 5′ nontranslated region (NTR) of the Jc1 (5′-CCU CAA AGA AAA ACC AAA CUU-3′) was used as a positive control siRNA^45^. siRNA targeting apoliprotein E (ApoE) (5′-TTC CTG GCA GGA TGC CAG GC-3′) was used as a positive control for HCV assembly^46^. siRNA transfection was performed using a Lipofectamine RNAiMax reagent (Invitrogen, Carlsbad, CA) according to the manufacturer’s protocol.

### Immunofluorescence assay

Huh7 cells grown on cover slides were washed twice with PBS and fixed with cold methanol at −20 °C for 10 min and then with cold acetone for 1 min. After three washes with PBS, fixed cells were blocked with 1% BSA in PBS for 1 h at room temperature. Cells were then incubated with a mouse anti-DR6 monoclonal antibody and a rabbit anti-NS5A antibody. After three washes with PBS, cells were incubated with either fluorescein isothiocyanate (FITC)-conjugated goat anti-mouse IgG or tetramethylrhodamine isothiocyanate (TRITC)-conjugated donkey anti-rabbit IgG for 1 h at room temperature. Cells were then counterstained with 4′,6-diamidino-2-phenylindole (DAPI) to label nuclei. After three washes with PBS, cells were analyzed using the Zeiss LSM 700 laser confocal microscopy system (Carl Zeiss, Inc., Thornwood, NY).

### Nuclear/cytoplasmic fractionation

Nuclear and cytoplasmic fractionation was performed as reported previously^47^. Briefly, Huh7.5 cells grown in 10 cm-diameter dishes were treated with various concentration of SN50. At 48 h after treatment, cells were washed twice with ice-cold PBS and collected into 1.5 ml tubes. After centrifugation at 13,000 rpm for 10 sec, cell pellet was resuspended in ice-cold 0.1% NP40 in PBS. Samples were triturated five times using a p1000 micropipette and centrifuged at 13,000 rpm for 10 sec. Supernatant was collected as a cytoplasmic fraction. The pellet was washed three times with ice-cold 0.1% NP40 in PBS and then was resuspended in RIPA buffer. Samples were sonicated three times for 30 sec and then was centrifuged at 13,000 rpm for 2 min to collect a nuclear fraction.

### Luciferase reporter assay

For a dual-luciferase reporter assay, Huh7.5 cells were cotransfected with a pRL-HL plasmid containing both the *Renilla* luciferase gene under the cytomegalovirus (CMV) promoter and the firefly luciferase gene under the control of the HCV IRES and the plasmid indicated in the figures together with the pCH110 reference plasmid^48^. At 48 h after transfection, cells were harvested, and then luciferase assays were performed as we described previously^49^.

### HCV pseudoparticle entry assay

HCV pseudoparticles (HCVpp) containing E1 and E2 glycoproteins derived from genotype 1a (H77) or genotype 2a (JFH-1), and vesicular stomatitis virus (VSV) pseudoparticles (VSVpp) were generated as previously described^50^. Briefly, HEK293T cells were transfected with a Gag-Pol (polymerase)-packaging plasmid, a glycoprotein-expressing plasmid, and a transfer vector encoding the firefly luciferase reporter protein by using polyethyleneimine (Sigma-Aldrich). Supernatants containing HCVpp or VSVpp were collected at 48 h after transfection. Cells were then infected with either HCVpp or VSVpp for 6 h. Cells were then replenished with fresh culture medium. At 72 h postinfection, cells were harvested, and luciferase activity was analyzed.

### Statistical analysis

Data are presented as means ± standard deviations (SDs). Student’s *t* test was used for statistical analysis. The asterisks in the figures indicate significant differences, as noted in the figure legends.

## Electronic supplementary material

Dataset 1
