# Supplementary material for: Hepatitis C Virus Exploits Death Receptor 6-mediated Signaling Pathway to Facilitate Viral Propagation
Source: Sci Rep. 2017 Jul 25;7:6445. doi: 10.1038/s41598-017-06740-9 (PMC5527075; doi:10.1038/s41598-017-06740-9)

**Hepatitis C Virus Exploits Death Receptor 6-mediated Signaling Pathway to Facilitate Viral Propagation**

**Trang T. D. Luong**^1^**, Giao V. Q. Tran**^1^**, Dong-Jo Shin**^1¤^**, Yun-Sook Lim**^1^**, Soon B. Hwang**^1^**^*^**

^1^National Research Laboratory of Hepatitis C Virus and Ilsong Institute of Life Science, Hallym University, Anyang, South Korea

**Supplementary Figure Legends**

**Figure S1** **Protein expression patterns of DR6 vary by cell types.** (A) Cell lysates collected from HEK293T, Huh7, and Huh7.5 cells were immunoblotted with an anti-DR6 monoclonal antibody. (B) HEK293T, Huh7, and Huh7.5 cells were transiently transfected with V5-tagged DR6 expression plasmid. At 48 h after transfection, cell lysates were immunoblotted with an anti-V5 monoclonal antibody.

**Figure S2 HCV infection upregulates DR6 mRNA level in primary human hepatocytes.** Primary human hepatocytes were infected with HCV Jc1 for 4 h. At the indicated time points after infection, total RNAs were extracted and DR6 mRNA levels were quantified by qRT-PCR. Data represent average from three independent experiments.

**Figure S3 NAC blocks NF-κB nuclear translocation.** Huh7.5 cells were either mock-infected or infected with Jc1 for 4 h. At 48 h postinfection, cells were either left untreated or treated with increasing amounts of NAC. At 48 h after inhibitor treatment, cytosolic and nuclear fractions were prepared and subjected to immunoblot analysis to determine NF-κB level. GAPDH and Lamin A/C were used as cytoplasmic and nuclear marker, respectively.

**Figure S4 NS4B upregulates DR6 via ROS production*.*** (A) Huh7.5 cells were transfected with either vector or Myc-tagged NS4B. At 48 h after transfection, cells were either left untreated or treated with increasing amounts of NAC. At 48 h after treatment, total cell lysates were immunoblotted with the indicated antibodies. (B) Huh7.5 cells were transfected with either vector or Myc-tagged NS4B. At 48 h after transfection, cells were either left untreated or treated with increasing amounts of BAPTA-AM. At 48 h after treatment, total cell lysates were immunoblotted with the indicated antibodies. (C) Huh7.5 cells were transfected with either vector or Myc-tagged NS4B. At 48 h after transfection, cells were either left untreated or treated with increasing amounts of NAC or BAPTA-AM. At 48 h after treatment, intracellular mRNA levels of DR6 were analyzed by qRT-PCR. Experiments were performed in triplicate. The asterisks indicate significant differences (***, P* < 0.01, ****, P* < 0.001) from the value for the control.

**Figure S5** **Overexpression or silencing of DR6 displayed no effect on cell growth.** (A) Huh7.5 cells were transiently transfected with V5-tagged DR6 expression plasmid for 24 h, 48 h and 72 h. At the indicate time points, cell growth was assessed by WST-1 assay. (B) Huh7.5 cells were transfected with the 20 nM of the indicated siRNAs for 24 h , 48 h and 72 h. At the indicate time points, cell growth was assessed by WST-1 assay. Experiments were performed in duplicate.

Supplementary Figure S1


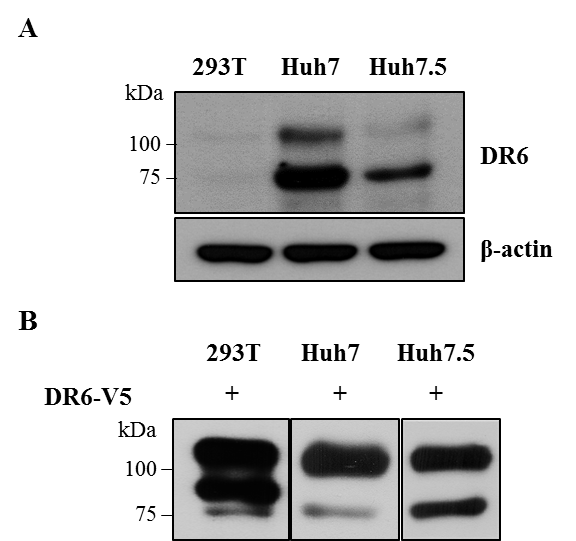


Supplementary Figure S2


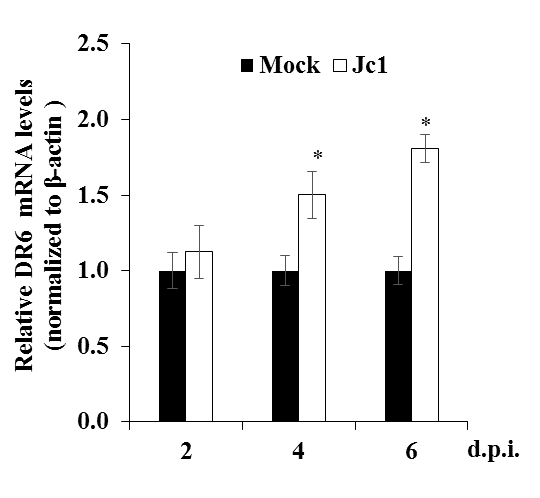


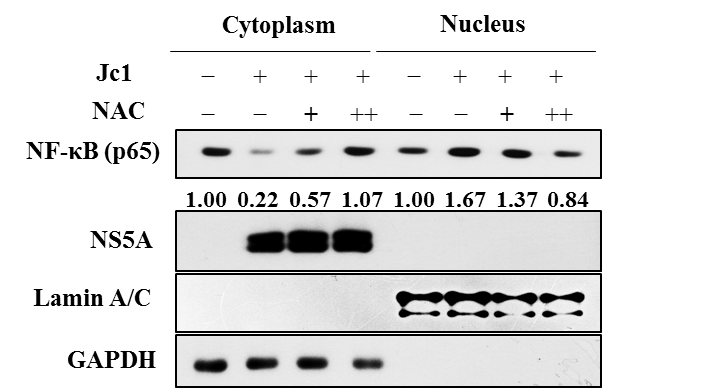


Supplementary Figure S3

Supplementary Figure S4


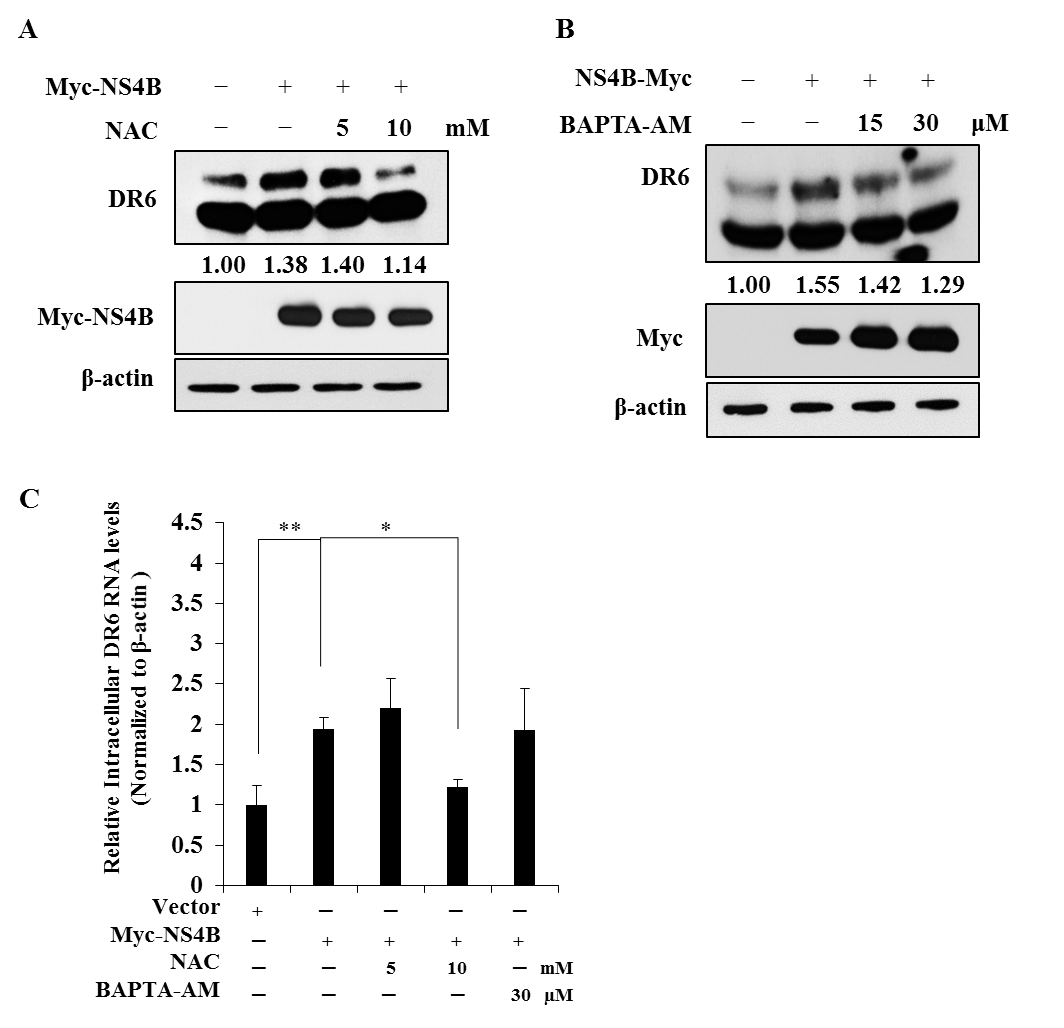


Supplementary Figure S5


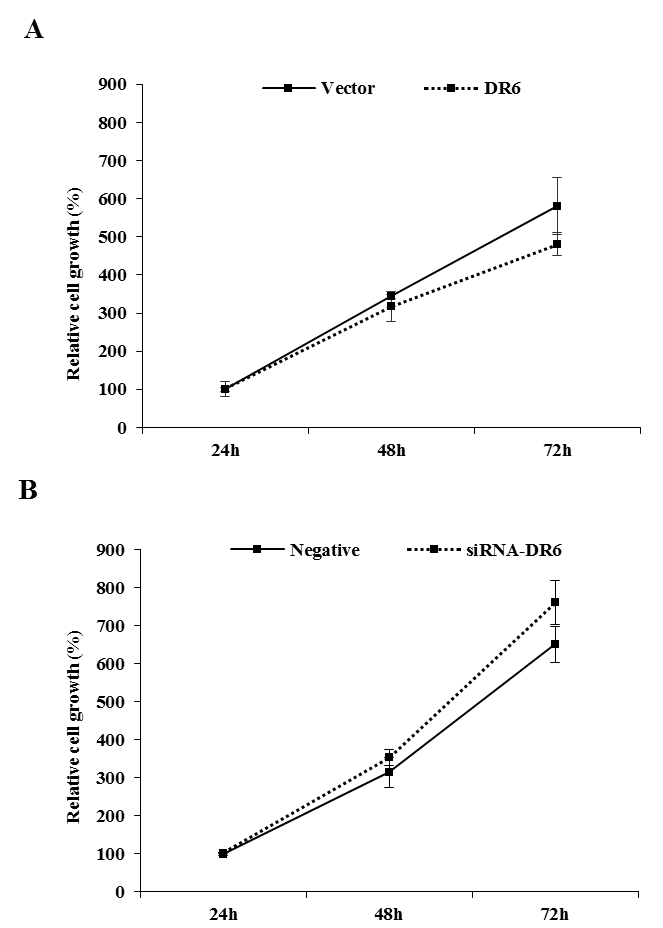

Supplement: Supplementary file 1 — Dataset 1 [file 41598_2017_6740_MOESM1_ESM.doc]
